# Conservative vs Surgical Treatment of Anterior Cruciate Ligament Rupture: A Systematic Review

**DOI:** 10.7759/cureus.56532

**Published:** 2024-03-20

**Authors:** Andreas Papaleontiou, Andréa M Poupard, Uday D Mahajan, Panteleimon Tsantanis

**Affiliations:** 1 Trauma and Orthopaedics, Queen Elizabeth Hospital Birmingham, Birmingham, GBR; 2 Trauma and Orthopaedics, University of Birmingham, Birmingham, GBR

**Keywords:** anterior cruciate ligament injury, systematic literature review, conservative vs surgical management, acl rehabilitation, acl repair, anterior cruciate ligament (acl)

## Abstract

Anterior cruciate ligament (ACL) injury is very common, especially in young athletic individuals who injure themselves during sports involving pivoting actions.

Management options include conservative management, which involves progressive physical therapy, educating the patient on how to prevent instability, and the use of a hinged knee brace. Surgical management involves reconstruction of the torn ligament using an autograft or an allograft and ACL repair where the torn ligament is affixed back to the tibia or femur. The choice of management depends on the severity of the injury, other injuries in associated structures, the level of fitness, and the athletic goals of the patient.

Many studies exist on the management choice of ACL injury, but no clear consensus prevails. This study will examine the effectiveness of conservative versus surgical management. A literature review will be performed to identify appropriate papers which compare and evaluate the two approaches.

A literature search for randomized controlled trials (RCTs) and cohort studies comparing the conservative to surgical management of ACL injury was conducted on PubMed, Scopus, and Web of Science. Patient eligibility criteria included individuals older than 15 with an isolated, recent ACL injury diagnosis via an appropriate clinical test, MRI, or arthroscopy. Studies were eligible if they were using appropriate surgical or conservative methods, as mentioned previously, and measuring results via appropriate scores, tools, and methods that will be presented below. The follow-up timeline would be from presentation time until at least two years.

Five papers were found to be eligible. Overall, these papers included 462 patients. Two studies measuring overall knee symptoms, function, and sports activities using the International Knee Documentation Committee Subjective Knee Form (IKDC) score found that the operated group had a significantly higher score. The other two studies measured overall knee function and health using the Tegner & Lysholm and Knee Injury and Osteoarthritis Outcome Score (KOOS) scores. In both papers, no significant difference was found between the two groups.

Regarding Tegner's activity score, only one paper had significant findings. A significantly longer period to return to sports activities was observed in the operative group. Stability was significantly higher in all papers in the operated group. Osteoarthritis was measured using different tools in each paper. Only one paper found a significantly higher risk in the operated group. Only one paper indicated significantly more complications in the operated group regarding side effects.

Overall, very few differences were observed between the two treatment groups. The most significant differences observed were the higher stability and the longer recovery period in patients undergoing surgery. Large RCTs following patients for enough time are needed to prove if surgical treatment offers significant benefits over conservative treatment.

## Introduction and background

The anterior cruciate ligament's (ACL) function prevents anterior tibial translation, internal tibial rotation, and knee hyperextension. Preventing these motions increases knee stability [1]. This is particularly important for athletes participating in sports involving pivotal motions, rapid deceleration, and quick change of direction [2].

ACL is one of the most injured ligaments, and its injury is often caused by a non-contact mechanism during sports. Patients are generally young and physically active individuals. The American Academy of Orthopaedic Surgeons classifies ACL injury into Grade 1, 2, or 3 sprains according to severity [3]. The grading system is presented in (Table 1).

**Table 1:** American Academy of Orthopaedic Surgeons ACL injury grading ACL: Anterior Cruciate Ligament Ref no- [3]

Grade 1	The ligament is stretched slightly, but the stability of the knee joint is not affected.
Grade 2	A stretch of the ligament to the point that it becomes loose, and this is also referred to as a partial tear.
Grade 3	The ligament is completely torn into two pieces, and the knee joint is no longer stable. This is the most common type of ACL injury.

Approximately 50% of ACL injuries occur in conjunction with damage to other structures in the knee, including the nearby ligaments, menisci, or cartilage on the surface of bone [2]. Diagnosing ACL injury is reached by history and physical tests such as Lachman, anterior drawer tests, and MRI [4].

Treatment

The treatment choice is based on the patient's symptoms, type of injury to the ligament, examination, the growth remaining in his or her growth plates, and activity goals [5-6]. 

Short-term complications of ACL rupture are decreased mobility, stability, pain, and risks of surgery if surgically treated. Long-term complications are osteoarthritis, instability, and risk of reinjury [7].

Conservative treatment options include progressive physical therapy and rehabilitation, educating the patient on how to prevent instability, and using a hinged knee brace [8]. Surgical options include replacement of ACL using patellar tendon autograft, quadriceps tendon autograft, hamstring tendon autograft, and allograft from the patellar tendon, Achilles tendon, semitendinosus, gracilis, or posterior tibialis tendon [5].

Non-surgical treatment is most appropriate for grade 1 injuries. Surgical treatment is recommended for individuals with a grade 3 or complete ACL tear [9]. Despite that recommendation, many factors contribute to the decision. Higher success rates of non-surgical management are observed amongst patients with partial tears and no instability symptoms, with complete tears and no symptoms of knee instability during low-demand sports, with low activity demands, and in children, as their growth plates are still open [10]. Therefore, active adult patients involved in sports or manual jobs are commonly considered for surgery.

Reasons for the need for the study

The right treatment choice is vital as it can minimize complications and improve the individual's quality of life. Complications of ACL rupture that must be considered are instability, stiffness, weakness of the knee, risks of the operation, graft rejection, and arthritis.

Current literature does not conclude whether operating on patients with an ACL injury is more beneficial than not operating. In addition, there is no clear consensus on whether surgery benefits certain patient groups more than others. Clear evidence-based guidance must be introduced to avoid unnecessary surgeries.

Aim

This literature review evaluates whether surgical management of ACL injury is superior to non-surgical treatment. 

Eligible studies must recruit patients older than 15 years old with a recently diagnosed isolated ACL rupture that underwent either surgical or non-surgical management. Surgical interventions are ACL replacement using autografts or allografts. Non-surgical interventions include physical therapy, rehabilitation, and patient education about instability prevention.

Outcomes will be measured by the potential differences in overall knee health, joint stability and function, development of osteoarthritis, and patient activity level, all taken at any time point since the intervention. Furthermore, the secondary aim will be to evaluate whether there are any differences in patient outcomes for surgical and conservative management between specific patient groups: professional athletes and regular individuals.

## Review

Methods

Literature Search Methods

A literature search was conducted in April 2020 in the following databases: PubMed, Scopus, and Web of Science. The search terms used were based on the population, intervention, comparison, outcomes, and study (PICOS) framework and the aim of the study [11]. The following search terms were used: ACL, Anterior Cruciate Ligament, Rupture, Injury, Reconstruction, Surgery, Repair, Non-surgical, Conservative, and Rehabilitation. The reference lists were reviewed for additional pertinent studies.

**Figure 1:**
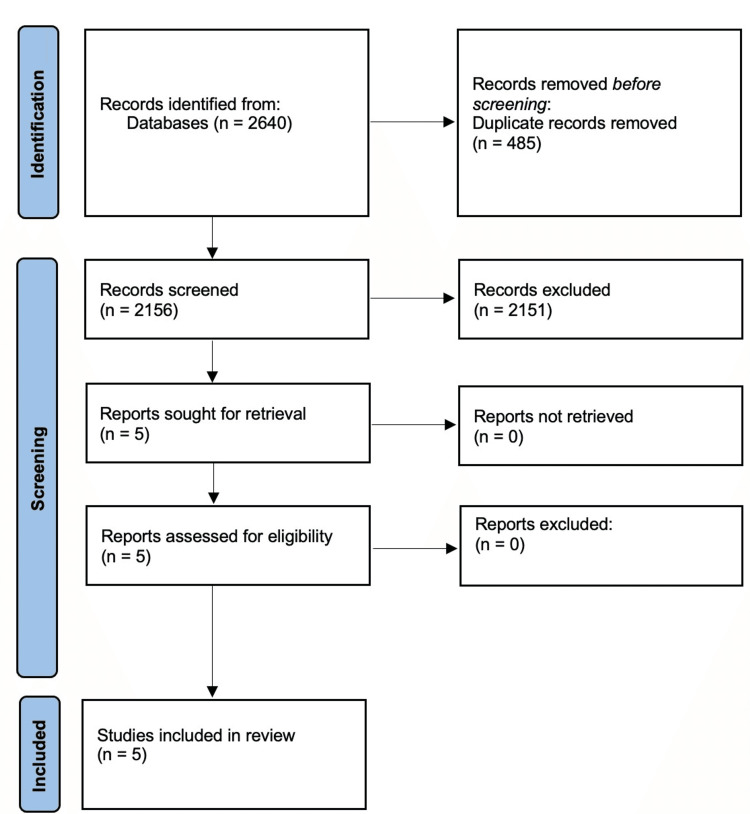
PRISMA 2020 flow diagram

Results were combined, and duplicates were removed as per Bramer et al. using the EndNote database represented in the PRISMA flow diagram (Figure 1) [12-14].

Study Eligibility Criteria

Randomized controlled trials (RCTs) and cohort studies were included in the search. In general, the papers selected must outline outcomes of surgical and non-surgical interventions for ACL rupture. The inclusion and exclusion criteria are outlined in (Table 2).

**Table 2:** Inclusion and exclusion criteria ACL: Anterior cruciate ligament

Inclusion criteria	Exclusion criteria
Randomised controlled trials, prospective and retrospective cohort studies	Only one management method e.g., only surgery or only non-surgical
Surgically and non-surgically managed patient cohorts	Multi-ligament knee injuries
Analysis of results for isolated ACL tears	History of previous ACL reconstruction
Follow-up outcomes reported for all patient cohorts	Reviews, systematic reviews, case reports, abstracts, conference proceedings
English language	Animal or cadaveric studies

Papers will be evaluated for their reliability, validity, sample selection process, outcomes measured, and methods of measuring them using the critical appraisal skills programme (CASP) appraisal checklist.

Patient Eligibility Criteria

Patients in studies that will be selected must have an ACL injury diagnosed by clinical examination, MRI, or arthroscopy. Treatment must be received within eight weeks of the injury. Studies involving or not separating patients with other injuries except the ACL injury will be excluded. Patients can be categorized into athletes in high physical-demand sports and non-athletes.

Conservative management was defined as progressive physical therapy and rehabilitation, educating the patient on preventing instability and using a hinged knee brace.

Surgical management accepted were the replacement of ACL using patellar tendon autograft, hamstring tendon autograft, quadriceps tendon autograft, and allograft from the patellar tendon, Achilles tendon, semitendinosus, gracilis, or posterior tibialis tendon.

Identification of Eligible Studies

Studies after deduplication were exported to Rayyan for title and abstract screening. Subsequently, the full text of relevant studies was assessed [15].

Outcomes Measured and Methods of Measuring Them

Outcomes can be separated into short and long-term. Short-term outcomes include function, activity, pain, instability, and stiffness. These are measured by the International Knee Documentation Committee Subjective Knee Form (IKDC) score [16], the Tegner Lysholm Knee Scoring Scale, and the Knee Injury and Osteoarthritis Outcome Score (KOOS) [17]. The details about what the scores account for are outlined in (Table 3).

**Table 3:** Scores measuring knee condition IKDC: International Knee Documentation Committee Subjective Knee Form; KOOS: Knee Injury and Osteoarthritis Outcome Score

Score:	Measurements:
IKDC	Knee symptoms, function, and sports activities [16]
Tegner Lysholm Knee Scoring scale	A patient-reported instrument that consists of subscales for pain, instability, locking, swelling, limp, stair climbing, squatting, and the need for support [17]
KOOS	Pain, other symptoms, function in daily living, function in sport and recreation, and knee-related quality of life [17]

Long-term outcomes include osteoarthritis, measured by radiological evaluation on the Kellgren and Lawrence score. The Kellgren and Lawrence score is a classification system of osteoarthritis with five grades of severity [18]. Additionally, function, instability, and activity will be evaluated in the long term using the same tools as in the short-term section. Reinjury and reoperation rates will also be measured. Eligible papers must measure these outcomes using the outlined scores and methods.

Results

The screening of databases produced 784, 462, and 1394 search results for Web of Science, PubMed, and Scopus, respectively. Deduplication yielded 2156 articles that were screened using Rayyan [15]. Five studies were selected after full-text review. Two papers were part of the same trial, with one paper presenting results after two years and the other after five years. Study characteristics are outlined in (Table 4) [9,19-22]. This systematic review followed the PRISMA guidelines: the literature search results and the appropriate papers' selection process (Figure 1).

**Table 4:** Study characteristics

Study	Time of intervention from injury	Study design	Gender	Patients	Average age	Follow-up period (average)
Tsoukas et al. [19]	Range: 4-6 weeks Median: 6 weeks	Prospective cohort study	M: 32; F: 0	32	32	10.3 years
Kessler et al. [20]	N/A	Retrospective cohort study	M: 68; F: 41	109	30.7	11.1years
Frobell et al. [9,21]	Less than 4 weeks	RCT	F: 32; M: 89	121	26	5 years
Sandberg et al. [22]	Within 2 weeks	Randomized prospective study	No data	200	28	33 months

Findings of the studies

Overall Knee Health and Function

Tsoukas et al. [19] found a significant difference in the mean values of IKDC scores between the two groups. Particularly, the ACL reconstruction group scored a mean value of 86.7, SD 6.5, while the non-operative group scored 77.5, SD 13.

Kessler et al. [20] found that the operative group had a significantly better IKDC score. Normal IKDC (A) was reported in 53% of the individuals in the ACL reconstruction group, while only in 14% of the conservative group.

Sandberg et al. [22] measured overall knee health using the Tegner and Lysholm score. No significant difference was found between the two groups. The operative group reported a score of 90, and the non-operative group scored 89.

Frobel et al. [21,9] measured pain, symptoms, function in daily activities and sports, and knee-related quality of life using the KOOS subscale score, which indicated no significant difference between the two groups in the first two years of follow-up. The pain was 87.2 and 87.7, symptoms 78.7 and 83.0, function in daily activities 93.5 and 94.7, function in sports 71.8 and 71.2, and knee-related quality of life 67.3 and 63.0 in the conservative and operative groups, respectively.

At five-year follow-up, KOOS4 mean change was 44.9 points in the conservative group and 42.9 points in the operated group. This difference was insignificant.

Knee stability was significantly higher in the operated group after five years. In the conservative group, 33% had a normal Lachman test, and 40% had a normal pivot shift. In the operated group, 76% had a normal Lachman test, and 76% had a normal pivot shift.

The pivot shift in Sandberg et al. [22] was positive in 61.8% of the non-operative group and 28.4% of the operative. Additionally, 13 non-operative patients and none operated patients reported giving way during exercise. Concludingly, stability was significantly higher in the surgically managed group.

Tegner Activity Score

Tsoukas et al. [19] reported that all patients returned to previous activity without fully restoring the pre-injury level. Activity level remained the same as the pre-injury level (seven) in the reconstruction group, while in the conservative group, it decreased from seven to five.

Kessler et al. [20] reported a Tegner score decrease from a pre-injury level of 0.48 points in the operative group and 0.55 points in the conservative group. The difference in activity level decrease was insignificant.

Frobell et al. [21,9] found that 44% of the operative group patients returned to preinjury activity level, while 36% of the conservatively treated patients returned to the same level two years after the injury. An insignificant difference between the two groups was found at the five-year follow-up.

Sandberg et al. [22] indicated that the time to return to sports activities was significantly higher in the operative group than in the non-operative group (24+/-8 vs 15+/-10 weeks).

KT1000 Knee Laxity Testing

In Tsoukas et al. [19], laxity, measured using a KT-1000 arthrometer with an applied force of 134N, was significantly higher in the non-operative group. The anteroposterior tibial translation was 1.5mm (SD 0.2) for the ACL reconstruction group and 4.5 mm (SD 0.5) for the non-operative group.

Kessler et al. [20] also reported a statistically significant difference in KT1000. The difference in anterior tibial translation between the healthy and affected knee was found to be 3.5mm in the reconstructive group and 5.7mm in the non-operative group.

Osteoarthritis

Tsoukas et al. [19] reported osteoarthritic radiological findings of grade c or d according to the IKDC scoring in 23.5% of the ACL-reconstruction group and 33.3% of the non-operative group. This difference was statistically insignificant.

Kessler et al. [20] used the Kellgren and Lawrence score to measure osteoarthritis. Osteoarthritis risk was defined as Grade II and above. Osteoarthritis incidence was significantly higher in the reconstructive group, with 45% of the patients having osteoarthritic findings of grade ii and above compared to only 24% in the conservative group.

In the Frobel et al. [9,21] trial, the KOOS4 score was used to measure osteoarthritis at baseline after two and five years. No significant difference between the two treatment groups was found from baseline. The reconstruction group had a mean score of 39.2, and the group with rehabilitation plus optional delayed ACL reconstruction had a mean score of 39.4. Results may be unreliable because 23 out of 59 patients in the conservative group underwent ACL reconstruction after 11.6 months on average from the initiation of the study.

At the five-year follow-up, 113 patients were scanned for osteoarthritis using a weight-bearing radiograph. No statistical significance between groups was found.

In the rehabilitation group, 12% had osteoarthritic findings on the tibiofemoral component and 8% on the patellofemoral components, while 11% of patients operated either early or later had findings in tibiofemoral and 23% on the patellofemoral component.

Early ACL reconstruction had 16% tibiofemoral osteoarthritis and 24% patellofemoral osteoarthritis, while the delayed optional reconstruction group had 7% and 15%, respectively.

Adverse Effects

Tsoukas et al. [19] reported no complications or revision surgeries in either group.

Frobel et al. [21,9] study found no significant difference in the serious adverse events between the two groups. Specifically, in the operative group, three ACL graft ruptures and one arthrofibrosis were reported, while in the delayed optional reconstruction group, only one ACL graft rupture occurred during the five-year follow-up.

Sanberg et al. [22] reported no complications in the non-operative group. In the operative group, complications included one septic knee arthritis, one hematoma, two deep vein thrombosis, and nine patients requiring postoperative mobilization of the joint. Additionally, swelling persisting for over one year was significantly higher in the operative group by 0.5cm.

Discussion

Overall, Tsoukas et al.'s [19] most important conclusion was that reconstruction gave better functional outcomes and laxity measurement in athletes in their 30s and 40s, having isolated ACL rupture.

Sandberg et al. [22] and Kessler et al. [20] findings further support Tsoukas et al. [19] conclusion by indicating that operated knees had decreased chances of a positive pivot shift test. Furthermore, non-operated patients' satisfaction with their ability to participate in physical activities decreased more with time than the operated group. Sandberg et al. [22] concluded that surgery produces a more stable knee for the first few years and that non-operated knees had a quicker increase in muscle power, range of motion, and function regain. Therefore, a patient with high physical demands would be a possible candidate for surgery.

Frobel et al. [9,21] concluded that there was no significant difference between the two groups after five years in pain, symptoms, knee-related quality of life, general physical or mental health status, daily activity function, function in sports, return to pre-injury activity level, radiographic osteoarthritis, or meniscus surgery. In this study, athletes were excluded. Therefore, this conclusion concurs with Tsoukas and Sanberg's conclusion, indicating that non-athletes might not benefit from surgery. A definitive conclusion can't be reached from this paper because 51% of conservatively treated patients had an operation during the follow-up time, decreasing the results' reliability.

Literature indicates a strong correlation between ACL injury and osteoarthritis. Nebelung et al. [23] showed an increased risk of high-level athletes with definitive unstable knee developing cartilage lesions over 20 years. Sherman et al. [24] and Neyret et al. [25] reported that chronic knee instability leads to cartilage degeneration. Newman et al. [26] cohort study found osteoarthritis in 51% of men and 41% of women with ACL injury after 12-14 years. Despite the evidence supporting the importance of an intact ACL in decreasing the risk of cartilage degeneration, the literature does not prove that reconstruction reduces the risk [9,22]. Tsoukas et al. [19] found no significant difference between the two treatment groups. Frobel et al. [21,9] agreed with these findings.

On the other hand, results from Kessler et al. [20] indicate a significantly higher risk in the operated group of developing osteoarthritis after an average of 11.1 years.

Fink et al. [27] found that participation in sports involving pivoting movements increased the risk of osteoarthritis in non-operated patients. Therefore, the rationale is that a surgical approach for active individuals, especially those involved in sports, may benefit them and decrease the risk of osteoarthritis. 

Overall, this study has identified the currently available RCTs investigating the effectiveness of surgery over non-surgical management. Results from these studies were analyzed and compared to answer this review's aim. Although some conclusions could be drawn, this study highlights the scarcity of data and a limited number of high-quality studies investigating the topic.

Future studies require a longer follow-up period to adequately observe the injury's and osteoarthritis's development.

Limitations

The limitations of this review are highlighted in (Table 5). 

**Table 5:** Limitations of this study

Limitations
Different length of follow-up in each study
Different ways of measuring outcomes were used in each study. Despite that, some common ways between some studies were used
Different surgical techniques and different surgical teams with the experience of the surgeon not specified
Rehabilitation protocols were not specified therefore, possible differences between studies
Only five papers were eligible, including only one RCT (published as two papers with different follow up), two cohort studies, and one prospective study
The muscle strength and pre-injury activity level of patients in some studies were not specified and were not accounted for. This potentially affects the outcome and the recovery
All papers had a mean age of between 25-35 years old, but Sandberg et al. included patients up to 61 years old, which possibly affects the comparability of the results
Search results were restricted to open-access articles

## Conclusions

This review observed very few differences between the surgical and non-surgical cohorts. The most significant findings involved higher stability and a longer recovery in surgery patients. Some evidence supporting better function and overall knee health in the operated patients was found; however, these findings were nonunanimous across the literature. Furthermore, individuals with high physical demands, e.g., athletes, benefited more from a surgical approach than the rest. Large RCTs following patients for enough time are needed to prove if surgical treatment offers significant benefits over conservative treatment.
